# Multiple Periapical Lesions Influence the Expression of TLR4/NF‐κB Pathway Components and the Development of Hepatic Injuries in Healthy and Chronic Alcohol‐Consuming Rats

**DOI:** 10.1111/iej.70104

**Published:** 2026-01-21

**Authors:** Karem Paula Pinto, Isabelle da Cunha Degani, Jenif Braga de Souza, Renata Heisler Neves, Luciana Brandão‐Bezerra, Luciana Moura Sassone, Emmanuel João Nogueira Leal da Silva

**Affiliations:** ^1^ Department of Integrated Clinical Procedures, School of Dentistry Rio de Janeiro State University (UERJ) Rio de Janeiro Brazil; ^2^ Laboratory of Experimental Surgery, School of Medical Sciences Rio de Janeiro State University (UERJ) Rio de Janeiro Brazil; ^3^ Romero Lascasas Porto Laboratory of Helminthology, Department of Microbiology, Immunology and Parasitology, Medical Sciences College (FCM) Rio de Janeiro State University (UERJ) Rio de Janeiro Brazil; ^4^ Departament of Endodontics Grande Rio University (UNIGRANRIO) Rio de Janeiro Brazil

**Keywords:** alcoholism, apical periodontitis, endodontics, hepatic necrosis, hepatic steatosis, liver

## Abstract

**Aim:**

To evaluate the impact of multiple apical periodontitis (AP) on the expression of TLR4/NF‐κB pathway components, proinflammatory cytokine levels, and development of hepatic injuries in rats with and without chronic alcohol consumption.

**Methodology:**

Thirty‐two rats were assigned to four groups (*n* = 8): Control, AP, Alcohol, and Alcohol+AP. The Alcohol and Alcohol+AP groups received 25% ethanol solution. Multiple AP were induced through pulp exposure of four molars for 28 days. Following euthanasia, the jaws and livers were collected. Micro‐computed tomography was used to confirm periapical lesions. Liver samples underwent histopathological analysis and ELISA assay to measure TLR4, NF‐κB, IL‐6, and TNF‐α levels. Histopathological evaluation was performed using hepatic stereology to assess hepatocytes, sinusoids, Kupffer cells, steatosis, leukocyte infiltrate, and necrosis. Statistical analysis was carried out using one‐way ANOVA followed by the Student–Newman–Keuls (*p* < 0.05).

**Results:**

Hepatic levels of TLR4 and NF‐κB were significantly higher in AP and Alcohol+AP groups compared to Control and Alcohol groups (*p* < 0.05). IL‐6 and TNF‐α were significantly elevated in all experimental groups compared to the Control group (*p* < 0.05), with higher levels observed in the Alcohol+AP group compared to the other groups (*p* < 0.05). Experimental groups showed a significant reduction in hepatocyte density compared to the Control group (*p* < 0.05), while sinusoidal volume was significantly reduced in the AP group compared to the Control group (*p* < 0.05). Hepatic steatosis was absent in the Control and AP groups and there was no significant difference in the percentage of steatosis between Alcohol and Alcohol+AP groups (*p* > 0.05). No significant differences were observed in the number of Kupffer cells among groups (*p* > 0.05) and leukocyte infiltrate was absent in all groups. Necrosis was significantly higher in the AP and Alcohol+AP groups compared to the Control and Alcohol groups (*p* < 0.05), with the Alcohol+AP group showing a higher percentage of necrosis compared to the AP group (*p* < 0.05). Hydropic degeneration, focal inflammatory infiltrates, and hepatocyte necrosis were observed in the AP and Alcohol+AP groups.

**Conclusions:**

Multiple AP led to elevated TLR4, NF‐κB, IL‐6, and TNF‐α levels and significant hepatic alterations including hepatocyte degeneration and necrosis. When combined with alcohol consumption, multiple AP exacerbated ethanol‐induced liver damage.

## 1 Introduction

Apical periodontitis is an inflammatory condition that affects the periapical tissues, primarily triggered by bacterial infection within the root canal system (Nair 2006). The pathogenesis of this condition begins with the bacteria colonisation and the release of virulence factors, such as lipopolysaccharides (LPS), which infiltrate the periapical tissues (Siqueira Jr and Rôças 2007; Wen et al. 2024). This process activates host immune signalling pathways, leading to the production and release of proinflammatory cytokines, including tumour necrosis factor‐alpha (TNF‐α) and interleukin‐6 (IL‐6) (Siqueira Jr and Rôças 2007). These cytokines, in turn, promote immune cell recruitment, stimulate osteoclastic activity, and contribute to progressive bone resorption (Graunaite et al. 2012). The persistent presence of bacterial toxins with an exacerbated immune response results in chronic inflammation, progressive tissue destruction, and ultimately, the formation of periapical lesions (Nair 2004; Wen et al. 2024).

Approximately half of the global adult population has at least one tooth affected by apical periodontitis, highlighting its relevance as a significant public health concern (Tibúrcio‐Machado et al. 2021). The presence of multiple apical periodontitis lesions has been linked to elevated serum levels of proinflammatory mediators, which can trigger immune responses, extending beyond the oral cavity and affecting distant organs and the overall health (Gomes et al. 2013; Zhang et al. 2016; Georgiou et al. 2019). Additionally, the spread of bacteria and pathogen‐induced virulent factors into the bloodstream can further activate systemic proinflammatory pathways (Segura‐Egea et al. 2015). A growing body of evidence suggests that apical periodontitis may contribute to the onset or exacerbation of systemic conditions, including diabetes (Segura‐Egea et al. 2015; Cintra et al. 2018), cardiovascular diseases (Caplan et al. 2006; Cotti et al. 2011; Segura‐Egea et al. 2015; Cintra et al. 2018), preeclampsia (Khalighinejad, Aminoshariae, Kulild, and Mickel 2017), renal diseases (Khalighinejad, Aminoshariae, Kulild, Sahly, and Mickel 2017), kidney diseases (Lamba et al. 2023), rheumatoid arthritis (Damiani et al. 2024), and metabolic syndrome (Sarmento et al. 2025).

Studies have also suggested a potential association between apical periodontitis and hepatic diseases (Castellanos‐Cosano et al. 2013; Grønkjær et al. 2016). Clinical research has identified a significantly higher prevalence of periapical lesions in liver transplant recipients (Castellanos‐Cosano et al. 2013) and cirrhosis patients (Grønkjær et al. 2016) compared to healthy individuals. Additionally, recent studies have reported that apical periodontitis is associated with elevated levels of hepatic transaminases (ALT and AST) (Bordagaray et al. 2024), increased hepatic cytokine expression (Xiao et al. 2023), morphological alterations in liver tissue and a reduction in liver antioxidant capacity (Ferraz et al. 2024), particularly when combined with chronic alcohol consumption (Ferraz et al. 2024). Despite these findings, the exact mechanisms underlying the relationship between apical periodontitis and hepatic dysfunction remain unclear.

Studies have demonstrated that both toll‐like receptor 4 (TLR4) and nuclear factor‐kappa B (NF‐κB) are upregulated in hepatic diseases (Gan et al. 2018; El‐Kashef and Serrya 2019). Lipopolysaccharide (LPS), a major component of the outer membrane of Gram‐negative bacteria, along with damage‐associated molecular patterns (DAMPs) released by tissue injury, plays a crucial role in triggering hepatic inflammation by activating TLR4 expressed on Kupffer cells (Soares et al. 2010). This activation stimulates signalling pathways, primarily through NF‐κB, leading to the release of proinflammatory cytokines and reactive oxygen species, which contribute to hepatic tissue damage (Soares et al. 2010; Wu et al. 2018). It is well established that periodontal disease can trigger hepatic inflammatory response via the TLR4/NF‐κB pathway (Yue et al. 2020; Yue et al. 2021; Bai et al. 2022). However, no studies have investigated whether apical periodontitis elicits a similar response. Therefore, the present study aimed to evaluate the impact of multiple apical periodontitis on hepatic injury in rats, both with and without chronic alcohol consumption, by assessing hepatic alterations, proinflammatory cytokine levels, and the expression of TLR4/NF‐κB pathway components. The null hypothesis tested was that multiple apical periodontitis would have no effect on hepatic TLR4 and NF‐κB expression, hepatic proinflammatory cytokines levels, or liver injury in rats, regardless of chronic alcohol consumption.

## 2 Materials and Methods

This manuscript complies with the Preferred Reporting Items for Animal Studies in Endodontology (PRIASE) guidelines (Figure S1) and the respective checklist set forth by the International Endodontic Journal.

### 2.1 Selection and Preparations of the Animals

This experiment was conducted in accordance with the ethical principles for animal experimentation adopted by the Brazilian College of Animal Experimentation (COBEA) and the guidelines for euthanasia practices of the National Council for Animal Experimentation Control (CONCEA). The certificate from the Animal Care and Use Ethics Committee (CEUA) of the Instituto de Biologia Roberto Alcântara Gomes (IBRAG/UERJ) was obtained (ID: 006/2021).

Thirty‐two male Wistar rats weighing between 180 and 230 g were used. The animals were housed in a climate‐controlled facility (21°C–25°C) under a 12‐h light/dark cycle. They were kept in plastic cages and had *ad libitum* access to a balanced diet and liquid consumption throughout the experimental period. Animal health and welfare were monitored daily by an experienced veterinarian, with particular attention to behavioural changes indicative of discomfort or pain. Environmental enrichment measures, including cage rearrangement and cognitive stimulation, were implemented to minimise stress and ensure stable health conditions during the study.

The animals were randomly assigned to four experimental groups (*n* = 8 per group):

- *Control*: no alcohol and no apical periodontitis lesions;
- *AP*: no alcohol and four apical periodontitis lesions;
- *Alcohol*: alcohol consumption and no apical periodontitis lesions;
- *Alcohol+AP*: alcohol consumption and four apical periodontitis lesions.

### 2.2 Alcohol Consumption

The Alcohol and Alcohol+AP groups were subjected to a 25% ethanol solution (Absolute Ethanol) as their drinking source. To facilitate adaptation, ethanol was introduced gradually, starting at a 5% concentration and increasing by 5% per week until reaching the final concentration of 25% (Pinto et al. 2020). The Control and AP groups received only filtered water.

### 2.3 Induction of Periapical Lesions

After 5 weeks of alcohol consumption, the animals in the AP and Alcohol+AP groups underwent pulp exposure to induce four periapical lesions in the first and second upper and lower left molars. The rats were anaesthetised by intramuscular injection of 0.1 mL of 10% ketamine hydrochloride and 0.05 mL of 2% xylazine hydrochloride per 100 g of body weight. Once anaesthetised, the pulps of the upper and lower left molars were exposed using a ¼ carbide bur at the mesial fossa of the occlusal surface (Damiani et al. 2024). The bur was inserted to a depth corresponding to its diameter, carefully avoiding furcation perforation. Following pulp exposure, the teeth remained open to the oral environment for 28 days (Damiani et al. 2024), allowing lesion development. During this period, alcohol administration continued according to the experimental protocol.

### 2.4 Euthanasia and Sample Collection

Twenty‐eight days after pulp exposure, the animals were euthanized via an overdose of anaesthetic (Pinto et al. 2020). Following euthanasia, the jaws and livers were carefully harvested and fixed in 10% buffered formalin for further analysis (Sarmento et al. 2025).

### 2.5 Microcomputed Tomography Assessment

The mandibles and maxillae were sectioned in half and the left sides were scanned in a SkyScan 1173 micro‐CT device (Bruker, Kontich, Belgium). The parameters for image acquisition were as follows: 1120 × 1120 matrix, 70 kV and 114 mA, with a 1‐mm thick aluminium filter, exposure time of 320 milliseconds, rotation step of 0.3° and 360° around the vertical axis, and the isotropic resolution at 17 μm. The images were reconstructed using the NRecon software (v1.6.1.0; Bruker) with the following parameters: 4 of ring artefact correction, 40% of beam hardening correction and 2 of smoothing for all images. Image processing and analysis were performed using the CTAn program (v1.6.6.0, Bruker). A calibrated evaluator assessed the presence of periapical lesions.

### 2.6 Hepatic Levels of TLR4, NF‐κB, IL‐6 and TNF‐α

Liver tissue samples (5 g) were homogenized in ice‐cold phosphate‐buffered saline (PBS) (Thermo Fisher Scientific, Waltham, MA, USA) at a ratio of 10 mL per gram of tissue. The homogenate was then centrifuged at 5000 × g for 15 min at 4°C. The resulting supernatant was collected and used to quantify TLR4, NF‐κB, IL‐6, and TNF‐α levels using ELISA kits (Mouse Monoclonal Antibody, Novus Biologicals, Littleton, CO, USA), following the manufacturer's instructions. Absorbance was measured at 490 nm using a microplate reader (iMark, Bio‐Rad Laboratories, Richmond, CA, USA). Analyte concentrations were determined from a standard curve generated with known concentrations of recombinant protein and were normalized to the total protein content of each sample. NF‐κB levels corresponded to total protein content measured in liver homogenates by ELISA. Given the nature of NF‐κB as a transcription factor, these measurements were interpreted as an indirect indicator of NF‐κB pathway engagement, rather than a direct assessment of its nuclear translocation or transcriptional activity.

### 2.7 Liver Histological Processing and Analysis

Liver specimens were dehydrated through a graded series of ethanol and xylene solutions and subsequently embedded in paraffin. The paraffin‐embedded tissues were sectioned using a Leica Biosystem RM2125 RTS microtome (Leica Biosystems, Wetzlar, Germany) to obtain 5‐μm thick sections. These sections were placed in a 36°C water bath, mounted on slides, and stained with haematoxylin and eosin (HE) (Sigma‐Aldrich, St. Louis, MO, USA) for histological evaluation under light microscopy. Two calibrated evaluators, blinded to the experimental groups, performed the assessments.

For stereological analysis, a digital imaging system equipped with a camera (Olympus BX53 microscope, Olympus Optical Co. Ltd., Tokyo, Japan) was used. Ten randomly selected microscopic fields per specimen were analysed at 400× magnification, and images were captured using ToupTek ToupView software (version 3.7.5962, Hangzhou ToupTek Photonics Co., China). A 36‐point test system (D36) was applied for quantitative analysis (Mandarim‐de‐Lacerda 2003), estimating the volume density (percentage) of hepatocytes, sinusoids, steatosis, Kupffer cells, leukocyte infiltrate, and necrosis. Additionally, a qualitative descriptive analysis of histopathological liver alterations was conducted.

### 2.8 Statistical Analysis

The sample size was estimated based on previous studies (Chen et al. 2021; Xiao et al. 2023). Assuming an effect size of 1.0, a significance level of 5%, and a statistical power of 95%, the sample size calculation indicated the need for 8 animals per group. The effect size represents the expected magnitude of the difference between groups relative to data variability, with a value of 1.0 corresponding to a large effect. Accordingly, a total of 32 male Wistar rats were included in the study.

Data were analysed using the GraphPad Instat software (GraphPad Software Inc., California, USA). Statistical analysis was undertaken with a significance level of 5% (*p* < 0.05). The normality test used was Shapiro–Wilk. Nonparametric data were analysed using the Kruskal–Wallis test followed by Dunn's test, while one‐way ANOVA followed by Student–Newman–Keuls test was used for parametric data.

## 3 Results

No animals died during the experiments, and no specific serious or other adverse effects were observed.

### 3.1 Microcomputed Tomography Assessment

**FIGURE 1:**
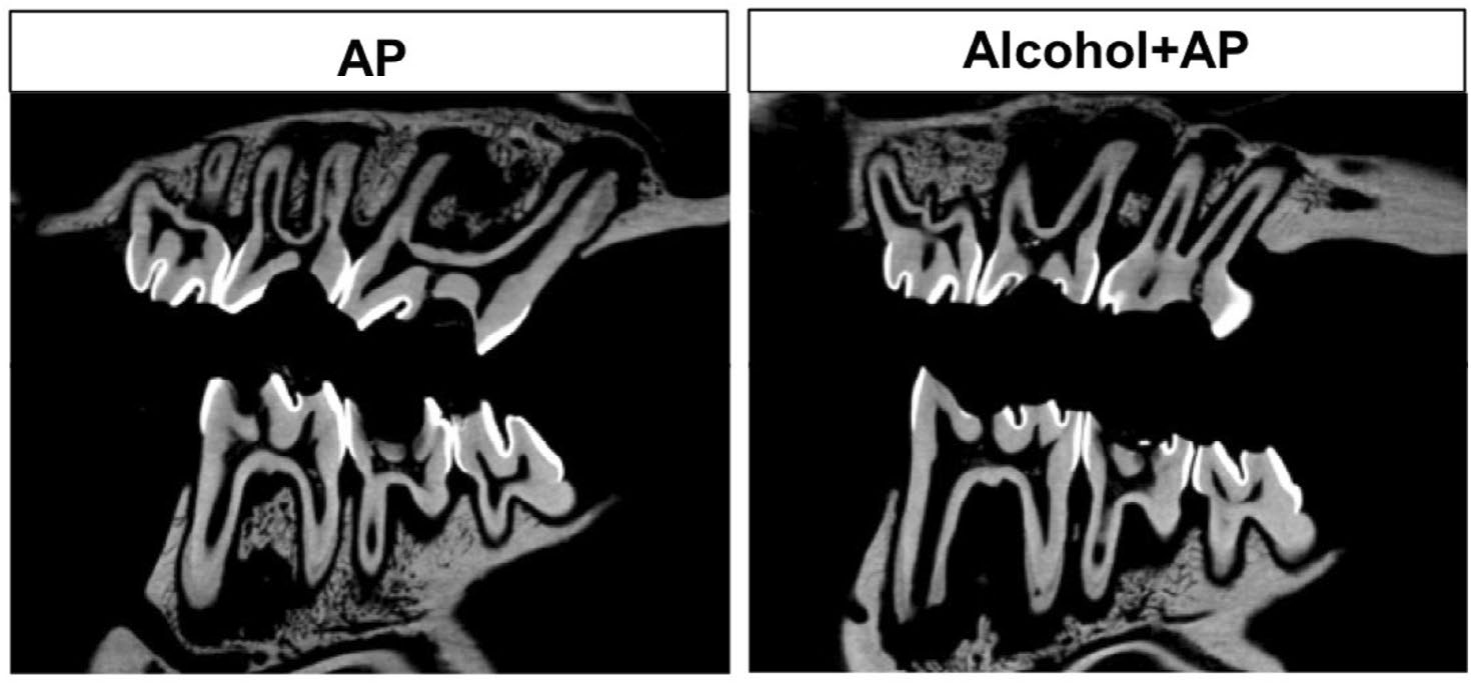
Representative micro‐CT images of the groups showing the 4 coronal access and the development of 4 periapical lesions.

All specimens developed multiple periapical lesions. Figure 1 shows the representative micro‐CT images of the groups showing the development of 4 periapical lesions.

### 3.2 Hepatic Levels of TLR4, NF‐κB, IL‐6 and TNF‐α

**FIGURE 2:**
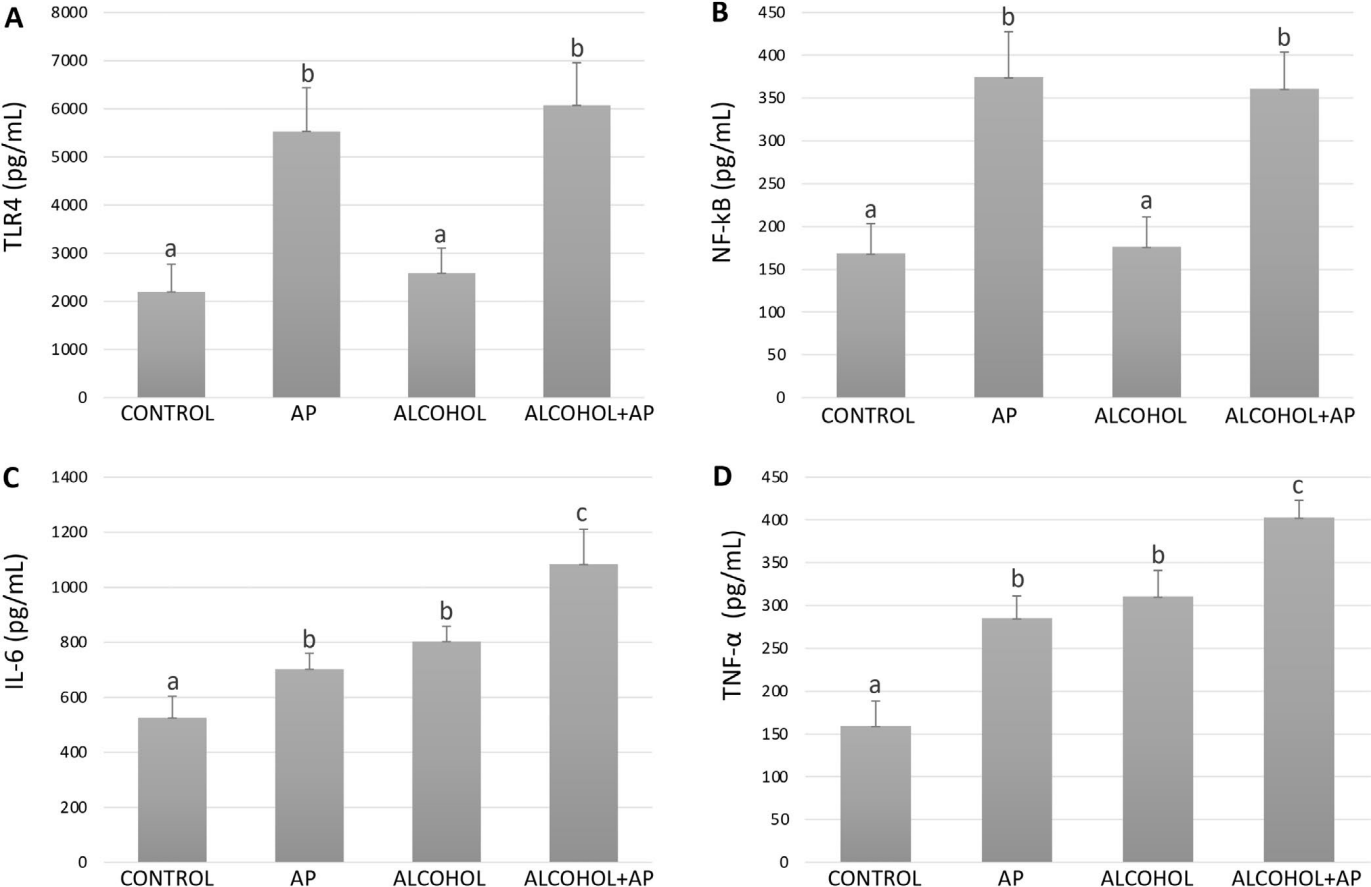
Hepatic levels of TLR4 (A), NF‐κB (B), IL‐6 (C), and TNF‐α (D) measured by ELISA assay. Different superscript letters indicate significant statistical difference between the groups (*p* < 0.05).

The ELISA assay results for hepatic TLR4, NF‐κB, IL‐6, and TNF‐α quantification are presented in Figure 2. TLR4 and total NF‐κB protein levels were significantly higher in the AP and Alcohol+AP groups compared to the Control and Alcohol groups (*p* < 0.05), while no statistically significant difference was observed between the Alcohol+AP and AP groups (Figure 2A,B), indicating that alcohol consumption did not further increase these markers beyond the effect associated with apical periodontitis alone. IL‐6 and TNF‐α levels were significantly elevated in all experimental groups compared to the Control group (*p* < 0.05), with the highest levels observed in the Alcohol+AP group (*p* < 0.05) (Figure 2C,D).

### 3.3 Liver Histopathological Evaluation

#### 3.3.1 Stereology

The stereological results are summarised in Table 1. Hepatocyte volume density was significantly lower in the AP, Alcohol, and Alcohol+AP groups compared to the Control group (*p* < 0.0001). Additionally, the Alcohol+AP group exhibited a 50% greater reduction compared to the AP group (*p* < 0.0001). However, no significant difference was observed between the Alcohol and Alcohol+AP groups (*p* > 0.05).

**TABLE 1:** Hepatic stereology of Wistar rats (mean ± SD).

Volume density (%)	Groups			
	Control	AP	Alcohol	Alcohol+AP
Hepatocytes	76.8 ± 3.8^a^	38.6 ± 14.9^b^	27.5 ± 8.7^bc^	19.3 ± 5.4^c^
Sinusoids	18.8 ± 3.9^a^	8.6 ± 4.9^b^	14.5 ± 3.2^ab^	13.5 ± 5^ab^
Steatosis	N.O.	N.O.	48.3 ± 17^a^	33.7 ± 10.2^a^
Kupffer cells	3.2 ± 1.2^a^	3.5 ± 1.2^a^	4.3 ± 2.2^a^	3.3 ± 1.4^a^
Leucocyte infiltrate	N.O.	N.O.	N.O.	N.O.
Necrosis	0.2 ± 0.4^a^	49 ± 16.2^b^	5.3 ± 6.9^a^	30.3 ± 19.5^c^

*Note:* Different superscript letters indicate significant statistical difference between the groups (*p* < 0.05).

Abbreviation: N.O., not observed.

Regarding sinusoidal volume density, the AP group showed a 54.2% reduction compared to the Control group (*p* = 0.0027). Hepatic steatosis was not observed in the Control and AP groups, and no significant difference was found between the Alcohol and Alcohol+AP groups (*p* > 0.05). Similarly, Kupffer cell volume density showed no significant differences among groups (*p* = 0.5713). Leukocyte infiltrate was absent in all samples, indicating no signs of liver infection. In contrast, necrosis volume density was significantly higher in the AP and Alcohol+AP groups compared to the Control and Alcohol groups (*p* < 0.0001), with the Alcohol+AP group presenting even higher necrosis levels than the AP group (*p* < 0.0001).

#### 3.3.2 Descriptive Analysis

**FIGURE 3:**
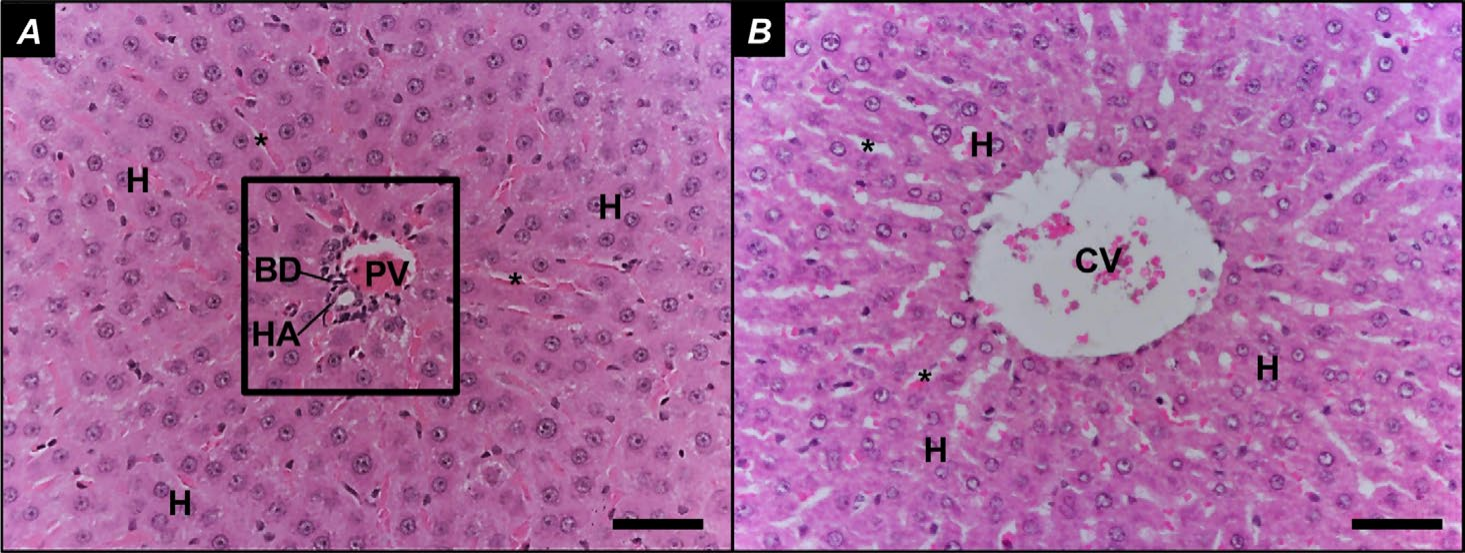
Representative images from ten randomly selected microscopic fields per specimen of the Control group. Liver parenchyma composed of cords of hepatocytes, permeated by sinusoids (A, B). In A, the portal space (composed of the bile duct, hepatic artery and portal vein) is represented and, in B, the centrilobular vein; BD, bile duct; CV, centrilobular vein; H, hepatocytes; HA, hepatic artery; PV, portal vein; Square, portal space; *: Sinusoids. Bars = 50 μm.

**FIGURE 4:**
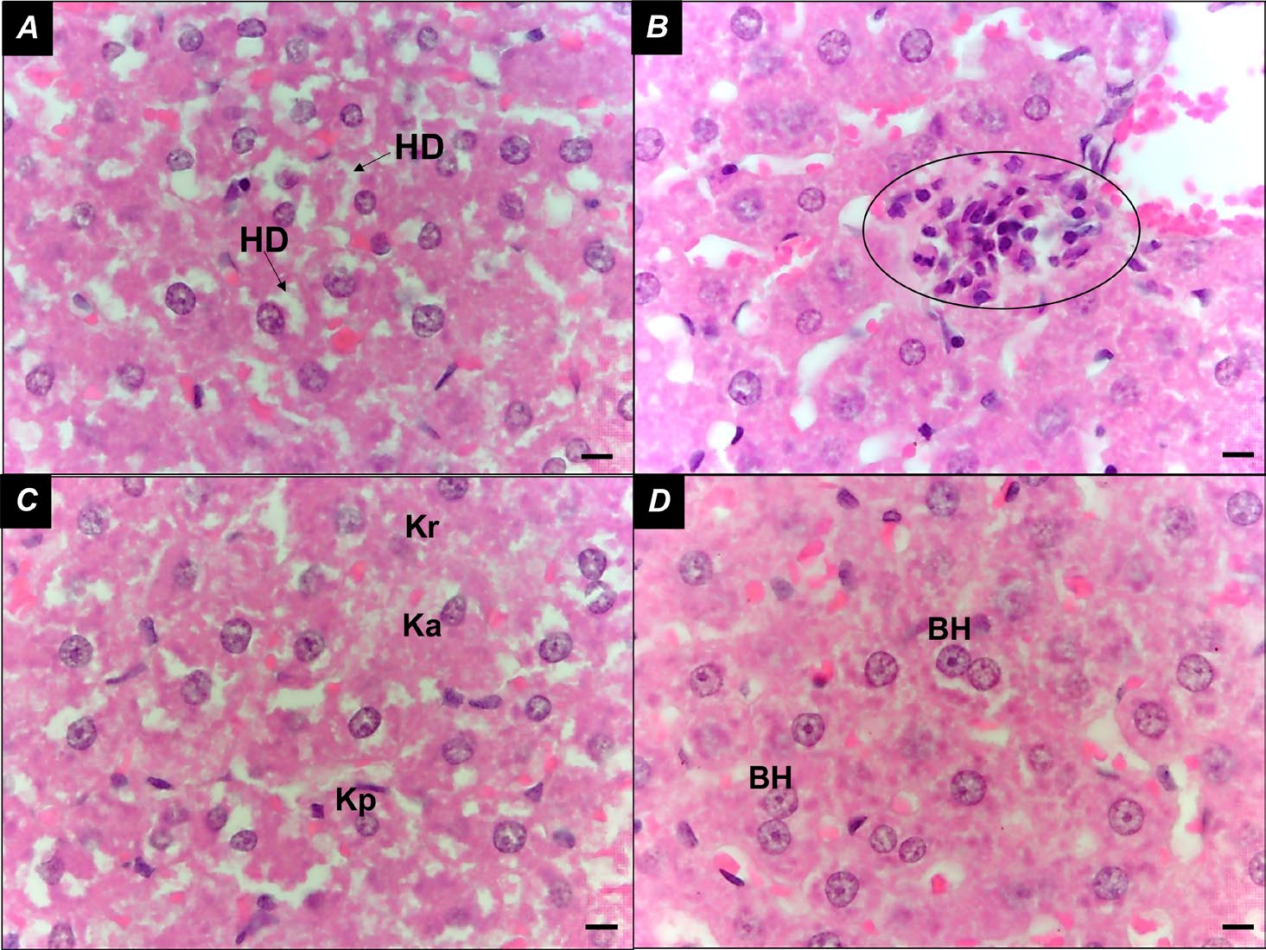
(A) Liver parenchyma with hydropic degeneration of liver cells, characterised by the presence of cytoplasmic vacuoles; (B) Focal inflammatory infiltrate; (C) hepatocyte necrosis—karyopyknosis, karyorrhexis and karyolysis; (D) binucleated hepatocytes. BH, binucleated hepatocytes; Ellipse, inflammatory infiltrate; HD, hydropic degeneration; Ka, karyolysis; Kp, karyopyknosis; Kr, karyorrhexis. Bars = 20 μm.

In the Control group, liver parenchyma appeared intact (Figure 3), with hepatocytes organised in cord‐like structures and sinusoids containing Kupffer cells within the capillaries. The portal space (Figure 3A) and centrilobular vein (Figure 3B) were clearly visible. In the AP group, hydropic degeneration of hepatocytes was observed, characterised by irregular cytoplasmic vacuolization (Figure 4A). Additionally, focal inflammatory infiltrates were present (Figure 4B), and liver cell necrosis was evident, displaying nuclear alterations such as karyopyknosis (chromatin condensation with nuclear shrinkage), karyorrhexis (nuclear fragmentation), and karyolysis (complete dissolution of nuclear components) (Figure 4C). The presence of binucleated hepatocytes was also noted (Figure 4D).

**FIGURE 5:**
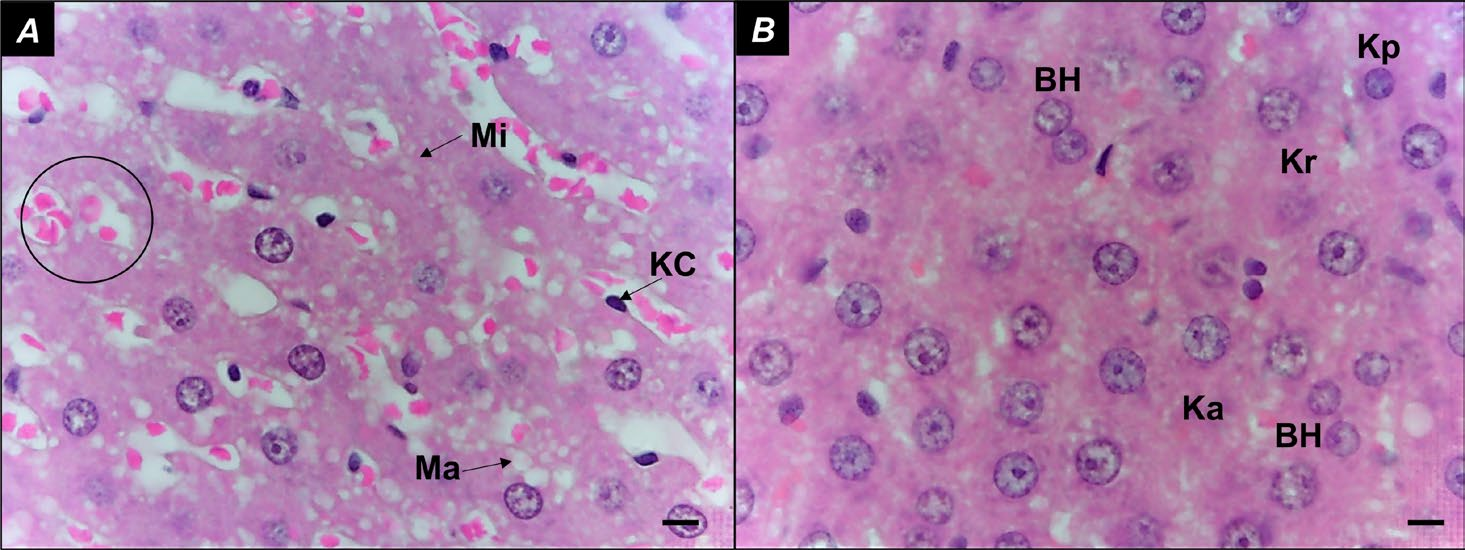
(A) liver parenchyma showing areas of alcoholic microsteatosis and macrosteatosis, Kupffer cells, and red blood cells retained within the sinusoids; (B) liver cell necrosis—karyopyknosis, karyorrhexis and karyolysis, and binucleated hepatocytes. Circle: Red blood cells retained within the sinusoids; BH, Binucleated hepatocytes; Ka, karyolysis; KC, kupffer cells; Kp, karyopyknosis; Kr, karyorrhexis; Ma, macrosteatosis; Mi, microsteatosis. Bars = 20 μm.

**FIGURE 6:**
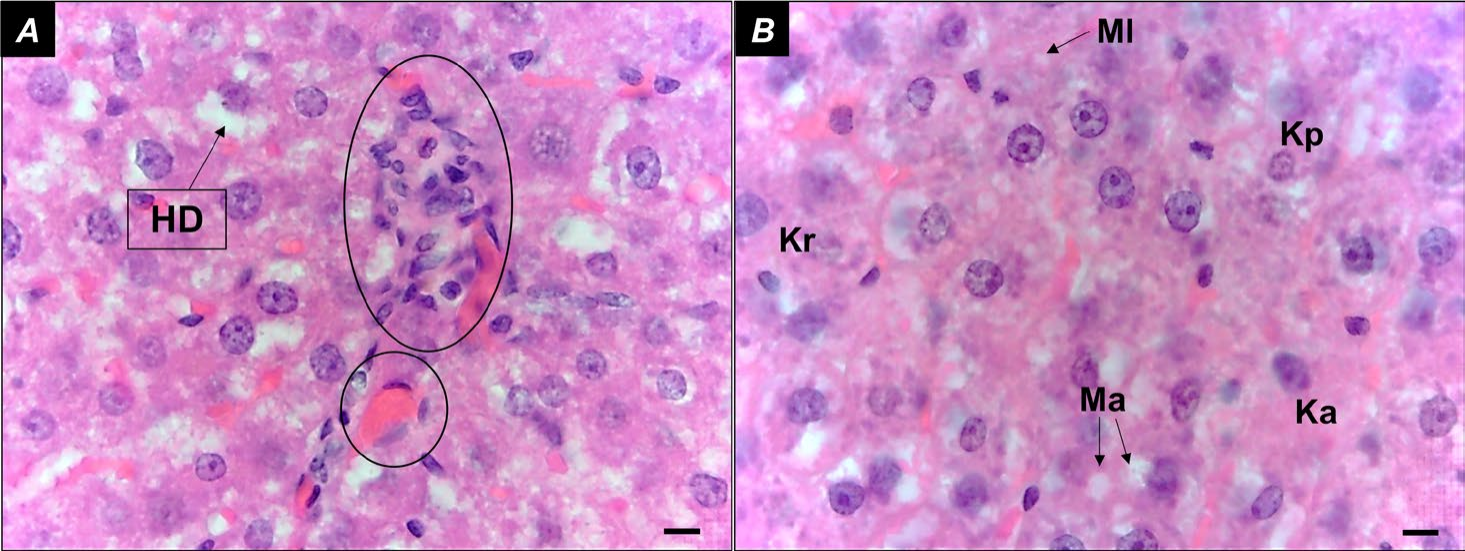
(A) liver parenchyma showing hydropic degeneration of liver cells, focal inflammatory infiltrate, and red blood cells retained in the sinusoids; (B) presence of areas of alcoholic microsteatosis and macrosteatosis, in addition to hepatocyte necrosis—karyopyknosis, karyorrhexis and karyolysis. Circle: Red blood cells retained within the sinusoids; Ellipse, focal inflammatory infiltrate; Ka, karyolysis; Kp, karyopyknosis; Kr, karyorrhexis; Ma, macrosteatosis; MI, microsteatosis. Bars = 20 μm.

In the Alcohol group, alcoholic microsteatosis and macrosteatosis were observed, along with Kupffer cell proliferation and red blood cells retained within the sinusoids (Figure 5A). Additionally, binucleated hepatocytes and necrotic liver cells exhibited characteristic nuclear changes such as karyopyknosis, karyorrhexis, and karyolysis (Figure 5B). In the Alcohol+AP group, focal inflammatory infiltrates were present, along with red blood cells retained within the sinusoids (Figure 6A). Similar to the AP group, hydropic degeneration of liver cells was observed (Figure 6A). Additionally, alcoholic microsteatosis and macrosteatosis were evident, along with widespread hepatocyte necrosis, characterised by karyopyknosis, karyorrhexis, and karyolysis (Figure 6B).

## 4 Discussion

This study aimed to investigate whether multiple periapical lesions could contribute, independently or synergistically with alcohol consumption, to the pathogenesis of hepatic disease in Wistar rats, evaluating TLR4/NF‐κB proinflammatory signalling. The null hypothesis that multiple apical periodontitis would have no effect on hepatic TLR4 and NF‐κB expression, hepatic proinflammatory cytokines levels, or liver injury, regardless of chronic alcohol consumption, was rejected as apical periodontitis increased hepatic levels of TLR4, NF‐κB, IL‐6, and TNF‐α, induced hepatocyte degeneration and necrosis, and exacerbated ethanol‐induced liver damage.

In this study, hepatic levels of TLR4 and NF‐κB were significantly higher in the AP and Alcohol+AP groups compared to the Control and Alcohol groups (*p* < 0.05). Previous research has demonstrated that periodontal disease can trigger a hepatic inflammatory response via the TLR4/NF‐κB pathway (Yue et al. 2020; Yue et al. 2021; Bai et al. 2022). However, this is the first study to investigate the impact of periapical lesions on hepatic TLR4 and NF‐κB levels. IL‐6 and TNF‐α levels were significantly elevated in all experimental groups compared to the Control group (*p* < 0.05), with the highest levels observed in the Alcohol+AP group (*p* < 0.05). These findings underscore the synergistic proinflammatory effects of alcohol and apical periodontitis on liver tissue. Additionally, they align with a recent study demonstrating that apical periodontitis induces liver inflammatory responses by increasing hepatic inflammatory markers, such as IL‐6 and TGF‐β1 (Xiao et al. 2023).

A decrease in hepatocyte density was observed in the AP, Alcohol, and Alcohol+AP groups, compared to the Control group (*p* < 0.05), with the most significant reduction occurring in the Alcohol+AP group. This finding further supports the synergistic detrimental effect of alcohol consumption and multiple periapical lesions on liver cells. Moreover, the AP group exhibited a significantly lower percentage of sinusoids than the Control group (*p* < 0.05). A recent study showed that apical periodontitis‐induced rats displayed increased liver sinusoidal dilation and reduced antioxidant capacity compared to control (Ferraz et al. 2024). Sinusoids are specialised blood vessels that play a crucial role in the exchange of nutrients, oxygen, and metabolites between the blood and liver cells, serving as the primary site for key liver functions (Koch et al. 2021). In contrast, alcohol‐exposed groups in this study did not show a significant difference in the sinusoid percentage compared to the Control group (*p* > 0.05), although red blood cells were retained within the sinusoids. This may be attributed to alcohol's ability to induce sinusoidal capillarization, a process in which sinusoids transform into systemic capillaries impairing bidirectional exchange between sinusoids and hepatocytes and leading to hepatocellular dysfunction (Mak et al. 2022).

In this study, hepatic steatosis was observed only in the groups submitted to alcohol consumption and was not impacted by apical periodontitis. However, the AP and Alcohol+AP groups showed a significant increase in necrosis (*p* < 0.05) when compared to the Control and Alcohol groups, with the Alcohol+AP group showing the highest percentage among all groups (*p* < 0.05). This finding corroborates a previous animal study that shows that apical periodontitis can cause irreversible liver damage, including hydropic degeneration and hepatocyte necrosis (Zhang et al. 2016). Indeed, hydropic degeneration was noted in the qualitative histopathological analysis of AP and Alcohol+AP groups in the present study. This is a common form of liver damage frequently observed in liver diseases and is caused by dysfunction of the sodium, potassium, and adenosine triphosphatase pump, leading to impaired water removal from the cell, which results in cellular swelling and may also progress to hepatocyte necrosis (Alpert and Hart 2016). The increased necrosis in the Alcohol+AP group highlights the synergistic detrimental effect of multiple apical periodontitis and chronic alcohol consumption on liver health, as chronic ethanol consumption also induces hepatocyte necrosis (Shojaie et al. 2020).

**FIGURE 7:**
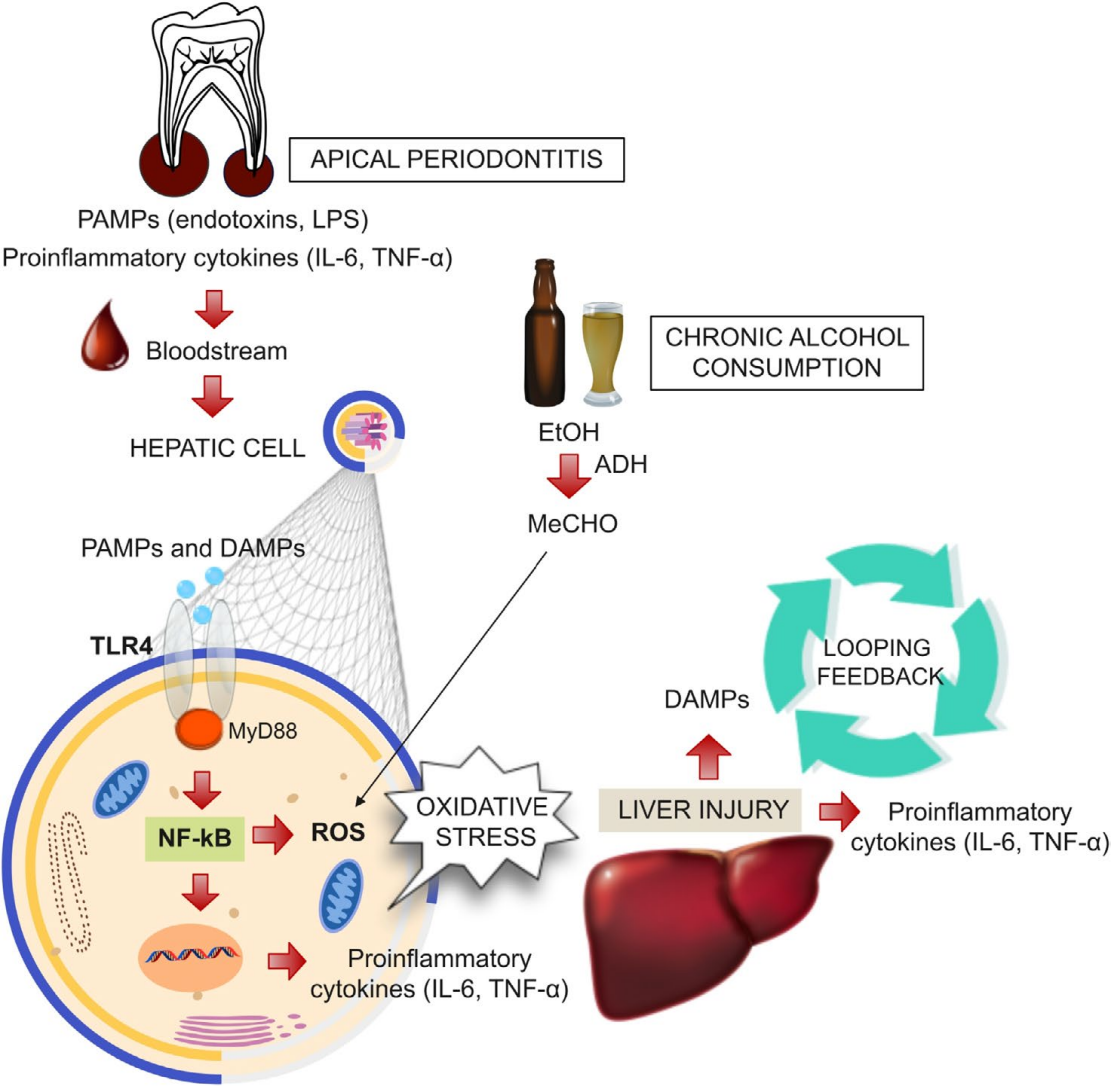
Hypothetical representative scheme of the effects of multiple apical periodontitis and chronic alcohol consumption on TLR4/NF‐κB–mediated liver injury. Bacterial LPS and proinflammatory mediators from apical periodontitis reach the liver and activate TLR4, which recognizes pathogen‐ and damage‐associated molecular patterns (PAMPs and DAMPs) and triggers Myeloid differentiation primary response 88 (MyD88)‐dependent NF‐κB signalling, increasing inflammatory cytokines and reactive oxygen species (ROS). Ethanol (EtOH) metabolism to acetaldehyde (MeCHO) by alcohol dehydrogenase (ADH) further enhances ROS production, promoting oxidative stress, liver damage, and DAMP release, which sustains a proinflammatory feedback loop.

The findings of this study suggest that multiple apical periodontitis may contribute to liver disease, primarily by increasing hepatocyte degeneration and necrosis, possibly through an exacerbated inflammatory response associated with TLR4/NF‐κB overexpression. When periapical lesions were combined with alcohol consumption, a synergistic effect was observed, further exacerbating liver damage. A hypothetical scheme illustrating how apical periodontitis and alcohol consumption may affect liver tissue through TLR4/NF‐κB overexpression is presented in Figure 7. These results highlight the importance of raising awareness among healthcare professionals and the public about the potential systemic impact of untreated periapical lesions, particularly in individuals with alcoholism. Since apical periodontitis is often asymptomatic, patients tend to delay treatment, leading to prolonged systemic exposure to inflammatory mediators (Cintra et al. 2021). Therefore, public health policies should prioritise the prevention, early diagnosis, and treatment of periapical lesions to mitigate their adverse effects on systemic health.

One of the key strengths of this study is its innovative approach, being the first to examine the impact of multiple periapical lesions on liver injury in both healthy and chronic alcohol‐consuming rats, with a focus on the potential involvement of the TLR4/NF‐κB proinflammatory signalling pathway. This novel perspective contributes significantly to understanding how periapical lesions may exacerbate liver damage. Additionally, this research introduces the quantification of TLR4 and NF‐κB levels into endodontic research, offering a valuable methodological asset for further studies on systemic inflammation. Another notable strength is the use of stereology for the quantitative analysis of liver damage, including assessment of hepatocytes, sinusoids, steatosis, Kupffer cells, leukocyte infiltration, and necrosis. This method is grounded in rigorous statistical principles, providing a reliable way to analyse three‐dimensional structures from two‐dimensional tissue sections (Mandarim‐de‐Lacerda 2003). Stereology is widely recognised as a highly effective tool for evaluating hepatic alterations and allows for precise estimations of critical parameters such as volume, surface area, and cell count, thereby enhancing the accuracy of liver damage assessment (Marcos et al. 2012; Moudi et al. 2018; Sandoval et al. 2019; Silva‐Veiga et al. 2020; Marcos et al. 2012). However, a limitation of this study was the inability to quantify the levels of endotoxins such as LPS and LTA, which are one of the key inducers of the TLR4/NF‐κB signalling pathway. These components are essential for understanding the full scope of the inflammatory response triggered by apical periodontitis. Additionally, the lack of an intervention group to evaluate whether endodontic treatment or pharmacological inhibition of the TLR4/NF‐κB pathway could mitigate liver damage represents another limitation. Future studies should investigate the microbiological effects of apical periodontitis on systemic organs, particularly focusing on the role of gut and oral microbiota dysbiosis in systemic inflammation. Furthermore, the use of oxidative stress markers and antioxidant defences could provide a more comprehensive understanding of the mechanisms involved in hepatic damage. Experimental approaches using inhibitors of the TLR4/NF‐κB pathway could help clarify the direct contribution of this signalling cascade to liver injury. Finally, clinical studies assessing the association between untreated periapical lesions and liver dysfunction in humans, particularly in individuals with chronic alcohol consumption, would be valuable in translating these findings to clinical practice.

## 5 Conclusion

Multiple apical periodontitis increased hepatic levels of TLR4, NF‐κB, IL‐6, and TNF‐α, leading to hepatocyte degeneration and necrosis. When combined with alcohol consumption, multiple apical periodontitis exacerbated ethanol‐induced liver damage.

## Author Contributions

**Karem Paula Pinto:** conceptualization, analysis, experimental procedures, writing, review (lead). **Isabelle da Cunha Degani:** analysis, experimental procedures, writing. **Jenif Braga de Souza:** experimental procedures, analysis. **Renata Heisler Neves:** analysis, experimental procedures; **Luciana Brandão‐Bezerra:** analysis, experimental procedures. **Luciana Moura Sassone:** conceptualization, analysis, experimental procedures, writing, review, and editing (lead). **Emmanuel João Nogueira Leal da Silva:** conceptualization, analysis, experimental procedures, writing, review, and editing (lead).

## Funding

This study was partially funded by CNPq and FAPERJ ‐ Fundação Carlos Chagas Filho de Amparo à Pesquisa do Estado do Rio de Janeiro, under grant No. SEI‐260003/013346/2024.

## Ethics Statement

The certificate from the Ethics Committee for the Care and Use of Experimental Animals (CEUA) of the Roberto Alcântara Gomes Biology Institute (IBRAG) of the Rio de Janeiro State University (UERJ) was obtained (006/2021).

## Conflicts of Interest

The authors declare no conflicts of interest.

## Supporting information


**Figure S1:** Flowchart developed in accordance with the PRIASE guidelines.

## Data Availability

The data that support the findings of this study are available from the corresponding author upon reasonable request.
