# Supplementary material for: Multiple Periapical Lesions Influence the Expression of TLR4/NF‐κB Pathway Components and the Development of Hepatic Injuries in Healthy and Chronic Alcohol‐Consuming Rats
Source: Int Endod J. 2026 Jan 21;59(5):863–72. doi: 10.1111/iej.70104 (PMC13065900; doi:10.1111/iej.70104)
Supplement: Supplementary file 1 — Figure S1: Flowchart developed in accordance with the PRIASE guidelines. [file IEJ-59-863-s001.docx]

**PRIASE 2021 Flowchart***

***From: Nagendrababu V, Kishen A, Murray PE, Nekoofar MH, de Figueiredo JA, Priya E, Jayaraman J, Pulikkotil SJ, Camilleri J, Silva RM, Dummer PM. PRIASE 2021 guidelines for reporting animal studies in Endodontology: a consensus-based development. Int Endod J. 2021 Jan 15. doi: 10.1111/iej.13477.** [**https://onlinelibrary.wiley.com/doi/10.1111/iej.13477**](https://onlinelibrary.wiley.com/doi/10.1111/iej.13477)

**For further details visit:** [**http://pride-endodonticguidelines.org/priase/**](http://pride-endodonticguidelines.org/priase/)
